# A CMOS-integrated terahertz near-field sensor based on an ultra-strongly coupled meta-atom

**DOI:** 10.1038/s41598-024-61971-x

**Published:** 2024-05-20

**Authors:** Alexander V. Chernyadiev, Dmytro B. But, Yurii Ivonyak, Kęstutis Ikamas, Alvydas Lisauskas

**Affiliations:** 1 CENTERA Laboratories, Institute of High Pressure Physics PAS, Sokołowska st. 29/37, 01-142 Warsaw, Poland; 2 Institute of Applied Electrodynamics and Telecommunications, Vilnius University, Saulėtekio av. 9, 10222 Vilnius, Lithuania

**Keywords:** Terahertz, Near-field sensor, Metasurface, Split-ring resonator, Coupled resonators, Sensors and biosensors, Metamaterials, Terahertz optics, Electrical and electronic engineering, Electronic devices

## Abstract

Recently, plasmonic-based sensors operating in the terahertz frequency range have emerged as perspective tools for rapid and efficient label-free biosensing applications. In this work, we present a fully electronic approach allowing us to achieve state-of-the-art sensitivity by utilizing a near-field-coupled electronic sensor. We demonstrate that the proposed concept enables the efficient implementation and probing of a so-called ultra-strongly coupled sub-wavelength meta-atom as well as a single resonant circuit, allowing to limit the volume of material under test down to a few picoliter range. The sensor has been monolithically integrated into a cost-efficient silicon-based CMOS technology. Our findings are supported by both numerical and analytical models and validated through experiments. They lay the groundwork for near-future developments, outlining the perspectives for a terahertz microfluidic lab-on-chip dielectric spectroscopy sensor.

## Introduction

In recent years, the terahertz (THz) part of the electromagnetic spectrum has proven to be an enabling platform for the emergence of a large variety of new research directions. One example is the field of metamaterial-based plasmonics. It involves a number of new phenomena such as spectral modulation, wavefront manipulation, polarization transformation, and the field of active metamaterials^1^. One of the most promising application areas of metamaterials in the THz frequency range is biosensing. This is due to several reasons: (1) Many biomolecules exhibit resonance features in the THz frequency range, making it a suitable range for their characterization; (2) Metasurfaces, with their ultra-thin nature, enable easy integration onto microchips, facilitating on-chip biosensing applications; and (3) The real-time monitoring capability of metasurfaces provides valuable insights into the kinetics of biological reactions. Another notable review^2^ provides a thorough examination of THz sensing techniques and compares them to established methods of quantitative and qualitative description of biomacromolecules. Until now, this field has been mainly addressed with fluorescence-based assays^3^. This conventional methodology provides extreme sensitivity and enables multiplexing versatility and real-time monitoring. On the other hand, fluorescent labeling techniques are labor-intensive, incur additional costs, and can contribute as an additional source of noise. Therefore, there is a high demand for alternative label-free analytical techniques^4^. In the search for alternatives, electromagnetic radiation frequencies ranging from very low frequencies^5^ to microwaves^6,7^ have been explored for single-cell characterization to a certain extent.

At terahertz frequencies, metamaterial-based approach^8^ has taken the lead, paving the way for integration toward a complete lab-on-chip device. Many of the aforementioned advantages of using metamaterials for sensing are highlighted in the recent review of terahertz biochemical sensors^9^. Several publications describe biosensing experiments using silicon-fabricated metamaterials^10,11^. Even though the authors used a terahertz time-domain spectroscopy system to perform measurements, it is an important step in the direction of integration nonetheless. Recent literature reports indicate that fully integrated electronic solutions for biosensing, dielectric spectroscopy, and near-field imaging functionality are progressively extending into the sub-THz and THz frequency range^12,13^. The current status of these developments for biomedical applications one can grasp from the following review^14^.

**Figure 1:**
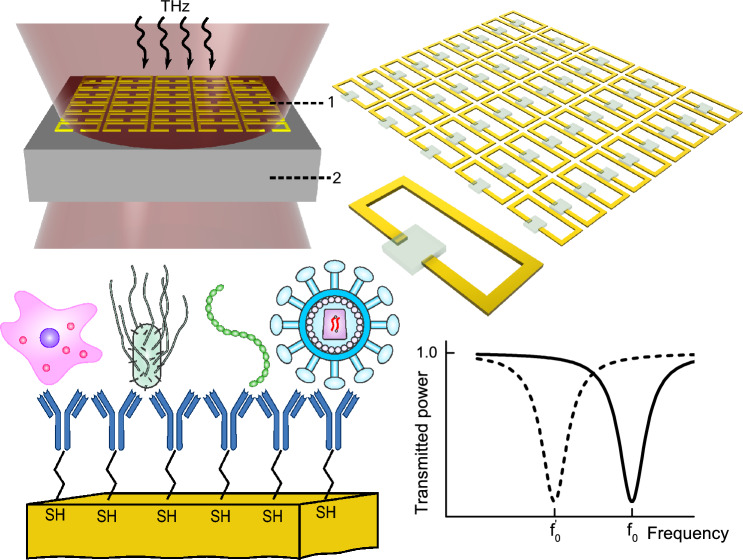
Biosensing based on resonance frequency shift. Top right: a metasurface of split-ring resonators covered with material. An idealistic scenario of homogeneous load distribution. Top left: a transmission experiment with a metasurface of resonators (1) fabricated on a substrate (2). Two measurements are undertaken: with and without material. Transmission spectra are collected in the far-field. Bottom right: a transmission spectrum recorded without material (solid line) shows an absorption feature at resonance frequency $f_0$, and a transmission spectrum recorded with material introduced (dashed line) is red-shifted with respect to the reference and features a resonance at $f'_0$. Bottom left: examples of analyte-specific sensing allowing to target a particular bacteria or virus achieved through conjugation mechanisms such as antibody-antigen binding, implemented after surface functionalization with thiol bonds.

That said, the most common approach to biosensing using metamaterials still involves covering the surface of resonating structures with a dielectric material, thereby changing the properties of the resonators and shifting their resonance frequency. Subsequently, a far-field sensing-based transmission experiment (see Fig. 1) is conducted to measure the frequency shift. At THz frequencies, concerted efforts have been made to enhance the sensitivity levels of resonance shift-based sensors to meet the standards of established methods in the field of biochemical diagnostics. The utilization of planar THz structures, such as waveguides and 2-D metamaterial arrays, is a common method in these articles^15,16^. For such structures, the standard metric for sensor comparison became the frequency shift (in units of Hz) per refractive index unit (RIU). In general, sensitivity depends on the quality factor (Q-factor) of the resonator and the line shape of the resonance. In the pursuit of higher Q-factors and better sensitivity, researchers have turned away from the traditional Lorentzian resonance curve toward more complex resonance behaviors, such as a Fano resonance^17^ or a toroidal resonance^18^. To further enhance sensor performance, optoelectronic nanomaterials have been incorporated, enabling the tunability of metamaterial response and thereby allowing control of the spectral properties of the sensor^19^. Furthermore, an effort has been made to develop methods that permit the probing of a single meta-atom^20^. Additionally, the applicability of such a sensor can be improved by finding a way to deliver small quantities of the material. Droplet-based microfluidics is a unique field that provides tools for rapid analysis of thousands of cells per run^21^.

**Figure 2:**
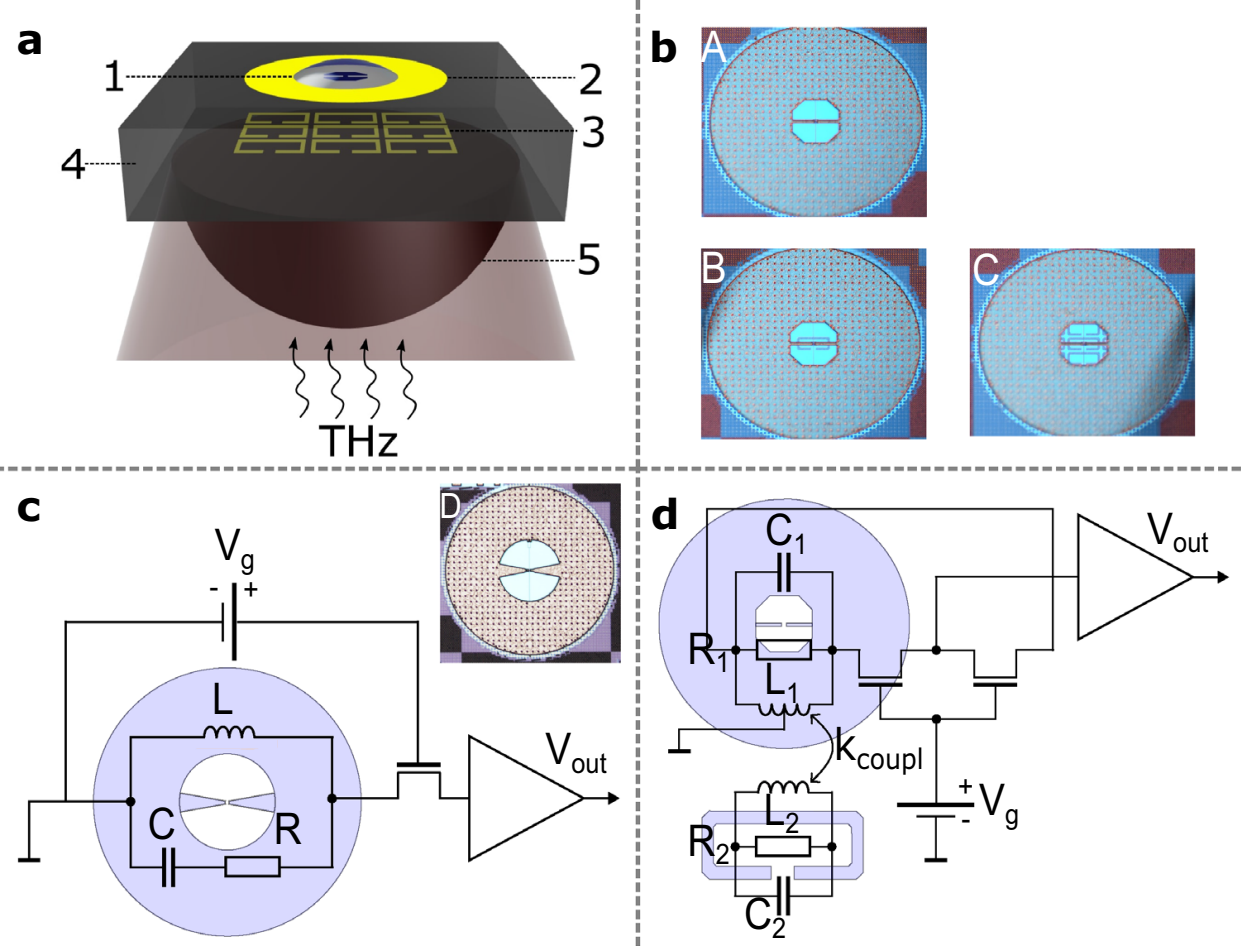
Schematic depiction of our concept of a near-field TeraFET detector-based sensor. (**a**) A 3-D illustration of a near-field sensor with an antenna coupled to a metasurface. 1 - a loading dielectric material, e.g. a droplet of water; 2 - a slot-dipole antenna; 3 - a metasurface of split-ring resonators; 4 - a silicon substrate; 5 - a silicon lens. (**b**) Micrographs of structures designed for 350 GHz. (**A**) - a resonant antenna, (** B**) - an antenna coupled to an SRR, (**C**) - an antenna coupled to a metasurface. (**c**) Micrograph of a resonant antenna designed for 235 GHz. An electronic schematic of the THz detection with the 235 GHz antenna. (**d**) An electronic schematic of the THz detection in a coupled resonators system.

In this article, we present a solution for a near-field sensor operating at sub-THz frequencies (Fig. 2). We demonstrate that it allows efficient realization and probing a single cell of an ultra-strongly coupled meta-atom. Our device has been implemented in silicon complementary metal-oxide semiconductor (CMOS) technology, and it utilizes field-effect-transistor-based (FET-based) THz power detectors^22^ as efficient near-field probes.

## TeraFET-based near-field sensor design

THz detection with FETs at frequencies above their cut-off ($f_T$) was demonstrated in the early 2000s^23^. Since then, there have been significant improvements in the design of TeraFET detectors, leading to many promising practical realizations in the fields of imaging^24,25^, spectroscopy^26,27^, and near-field imaging^28^. A monolithically integrated antenna is necessary for the efficient delivery of optical power from free space to the FET terminals^29^.

For this study, two different slot-dipole antennas^28^ were designed and implemented in the 180 nm Taiwan Semiconductor Manufacturing Company (TSMC) silicon-based CMOS process. One was designed to have resonance at 350 GHz with a roll-off at higher frequencies (structure A in Fig. 2b), while the other had resonance at 235 GHz with an almost flat response over a wide frequency range (structure D in Fig. 2c). Both antennas have the same outer diameter of the ring, i.e. 452 µm. As antenna D was designed for lower frequencies it features a larger slot (a circle of 164 µm diameter) compared to the slot of antenna A (a square aperture with a side length of 100 µm and 25$\times$25 µm chamfered edges). Furthermore, the transmission lines design is different in two antennas. Antenna A maintains constant width of metallization, equal to 6 µm, whereas in the case of antenna D, the width was varied from 32 µm to 6 µm providing a more broadband characteristic. The gap between the transmission lines was the same for both antennas, equal to 4 µm. Due to the differences in design and hence in characteristics, different analytical description was adopted for two antennas, which is highlighted in Fig. 2c,d and the second part (S.2) of the Supplementary information. Thanks to the multiple-layer metal-dielectric architecture, CMOS technologies provide ample freedom to explore metamaterial-based solutions for field-effect transistors in THz applications. Therefore, we designed a 350 GHz split-ring resonator (SRR) with an area of $70\times 30$ µ$\tt m^2$, a gap of 10 µm, and metallization width of 5 µm. Featuring the same resonance frequency as the 350 GHz antenna, the SRR electromagnetically couples to it. Thus, we investigated the coupling of vertically-oriented resonators and their sensitivity to changes in surrounding dielectric properties. The vertical arrangement provides unique versatility^30^ and guarantees stronger coupling than the case of lateral arrangement. This superiority arises from the considerably smaller metal thickness relative to the other two physical dimensions, enabling a significantly stronger interaction. In our case, the antenna and the SRR were vertically separated by 6.52 µm, being integrated into the top and bottom metal layer of the back end of line, respectively. In the described CMOS process aluminium metal layers were formed.

The antenna is situated in a metal layer which is the closest one to free space, farthest away from the substrate. Therefore, it is sensitive to the dielectric load applied to the near-field of the gap. The spectral change in the impedance characteristic of a single antenna/antenna coupled to an SRR is directly translated into the FET’s rectified signal alteration. Hence, a TeraFET detector with an antenna loaded with a dielectric material will give a signal different from a detector with no material. Thus, the near-field dielectric properties of test materials can be probed by the far-field THz radiation applied from the substrate side. Particularly, in Fig. 2, we present a visualization of the concept of near-field detection by a coupled resonators-based TeraFET detector. Fig. 2c,d show the terahertz detection mechanism in two different schemes: a stand-alone antenna implementation and a coupled resonators implementation, in which an antenna is coupled to an SRR. In the first case, the antenna is connected to a single NMOS transistor^31^ in a way that enables asymmetric power coupling into the channel^22^. In the second case, the antenna is connected to a couple of NMOS transistors placed in series^32^.

**Figure 3:**
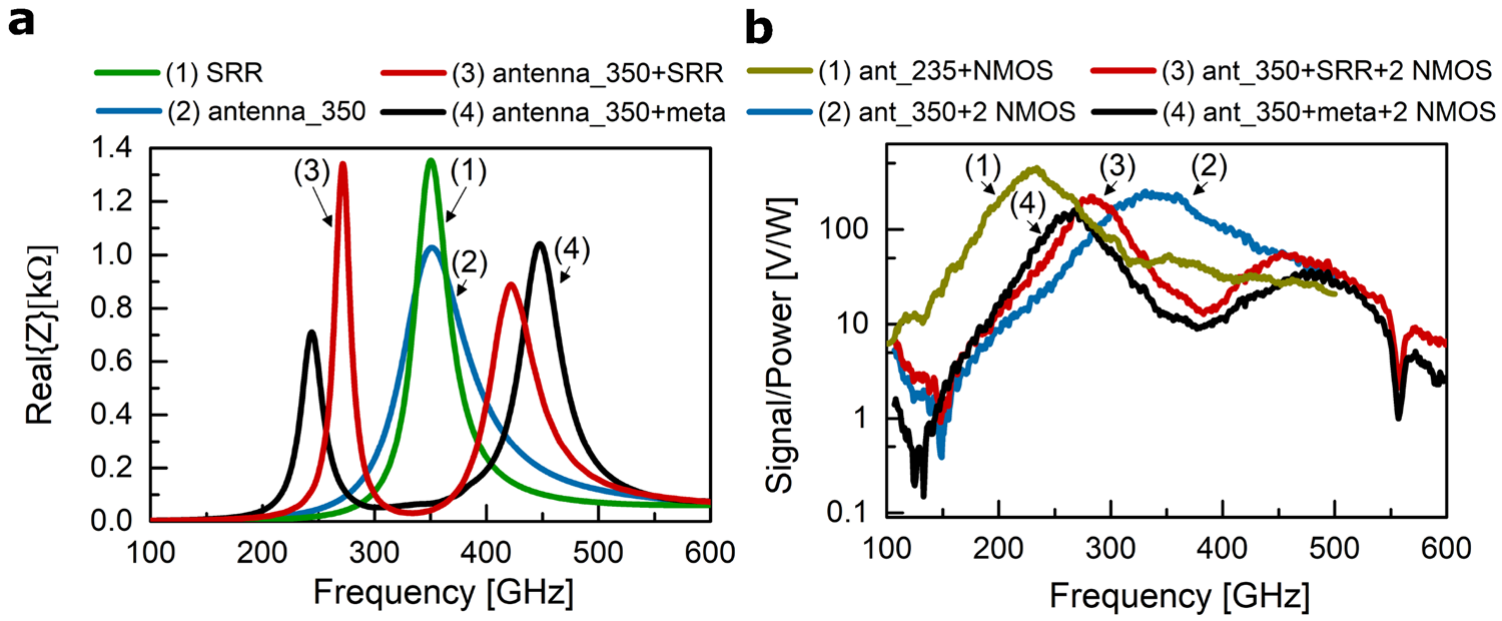
Detection: simulation and experimental results of both coupled resonator-based systems and stand-alone detectors. (**a**) Spectrum of the real part of the impedance of an antenna, an SRR, an antenna coupled to an SRR, an antenna coupled to a metasurface obtained from simulations. (**b**) Spectral response of a stand-alone detector designed for 350 GHz, of a detector with the 350 GHz antenna coupled to an SRR, of a detector with the 350 GHz antenna coupled to a metasurface, and a stand-alone detector designed for 235 GHz. The dark yellow curve is the characteristic of the design shown in Fig. 2c. The blue, red, and black curves are characteristics of the design presented in Fig. 2d.

The finite element method of the CST Microwave Studio software package was utilized to simulate the electromagnetic properties of the antennas and split-ring resonators that form a single meta-atom cell. The real part of the antenna’s impedance and the SRR’s impedance are presented in Fig. 3a. Both display resonance at 350 GHz. When coupled, two resonance modes appear: one at 274 GHz and another at 425 GHz. The coupling strength can be estimated from Eq. (1). It is equal to 0.43.

1 $$\begin{aligned} \nu = \frac{\Delta \omega }{\Omega }, \end{aligned}$$

where $\Delta \omega$ is the resonance frequency difference between the two modes after the splitting, and $\Omega$ is the common resonance frequency of the stand-alone resonators.

The last curve (black one) in Fig. 3a is plotted for a slot-dipole antenna coupled to a metasurface of 45 split-ring resonators. The separation distance between the two adjacent SRRs is the same in two orthogonal directions, i.e. 5 µm. Corresponding resonance modes are split even further apart - one at 244 GHz, another at 448 GHz. Hence, a normalized coupling strength of 0.58 is achieved. Therefore, we demonstrate that an equivalent situation for ultra-strong coupling can be realized at room temperature and cost-efficient fabrication technologies. The reader is referred to the second part (S.2) of the Supplementary information for the impedance analysis of uncoupled and coupled resonators in terms of equivalent circuits. This simple and practical approach provides valuable insights into designing resonators for specific frequencies along with understanding coupling effects via mutual inductance and mutual capacitance parameters. Having a semi-analytical model for coupled resonators proves to be advantageous for the description of the antenna load, which in turn allows us to quantitatively explain the sensing mechanism. The details of this method are presented in the third part (S.3) of the Supplementary information.

The numerical predictions of the structures’ resonance frequencies and the resonance splitting were verified in the experiment. In Fig. 3b, the rectified voltage response of detectors based on different structures is shown. The spectra were obtained with a widely tunable continuous-wave photomixer-based terahertz radiation source (part of a commercial Frequency-Domain Terahertz Spectroscopy System, TeraScan 1550, TOPTICA Photonics AG). A gate bias of 0.6 V was applied to the FET throughout the measurement, which is the optimal operating point for an NMOS transistor in a 180 nm technological process in terms of signal-to-noise ratio. One detector with an integrated slot-dipole antenna exhibits a resonance peak at 350 GHz, and another one at 235 GHz. The 350 GHz antenna coupled to a split-ring resonator displays two clear resonance frequencies: at $\approx$ 283 GHz and at $\approx$ 453 GHz. When the antenna is coupled to a metasurface of 45 SRRs, the resonance peaks shift to $\approx$ 267 GHz and $\approx$ 479 GHz. The experimentally determined coupling strength is 0.49 and 0.61, respectively. The 235 GHz detector shows the best responsivity values, but the coupled resonator-based detectors reveal their potential as a better near-field sensor at the limit of minute sample concentrations. During our research on stand-alone and coupled resonators, we arrived at an important conclusion: for large variations in the dielectric properties of a material, the stand-alone antenna is more sensitive than an antenna coupled to another resonator. On the other hand, coupled resonators are more sensitive to smaller variations in the near-field permittivity. The relevant results and analysis are provided in the third part (S.3) of the Supplementary information.

## Validation of the near-field sensing approach

**Figure 4:**
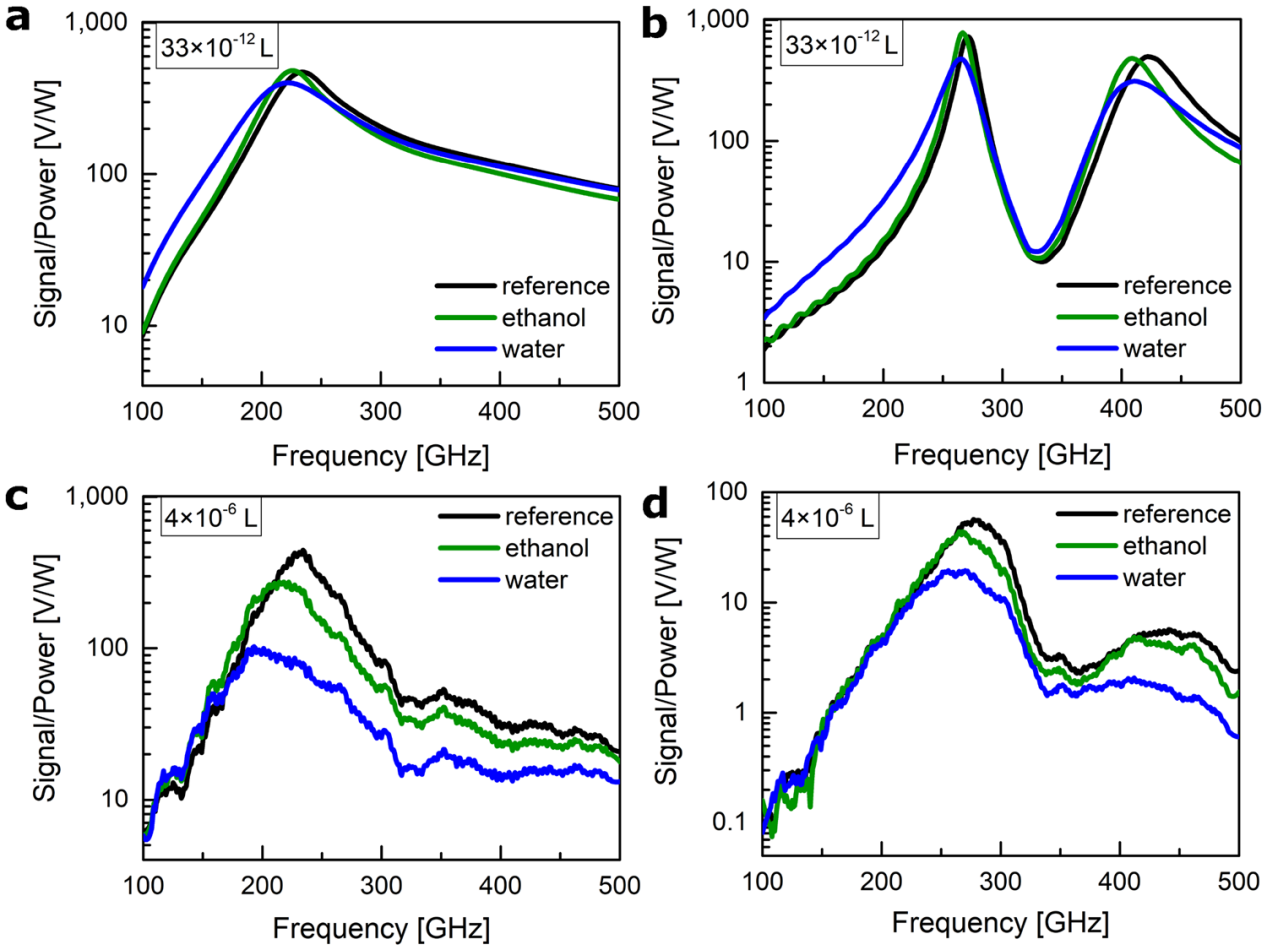
Sensing: simulation and experimental results for both a coupled resonators-based detector and a stand-alone detector. (**a**,**b**) Simulation. (**c**,**d**) Experiment. (**a**,**c**) Detector with a stand-alone antenna. (**b**,**d**) Detector with an antenna coupled to an SRR.

For the experimental validation of the applicability as a sensor, we concentrate on the 235 GHz antenna-based detector and the coupled resonator-based detector (350 GHz resonance antenna coupled to an SRR) loaded with water and ethanol. In Fig. 4 the left column represents the stand-alone detector, while the right column stands for the coupled resonators-based detector. An additional step of electronic circuit simulation in the commercial software package Advanced Design System from Keysight Technologies company was included in the numerical predictions to translate the changes in the antenna’s impedance (Fig. 3a) to the overall detector’s voltage response (Fig. 4a,b). An explanation to the question of why the simulations predict the two resonance modes of the coupled resonators to be almost equal (Fig. 4b), but the experiment shows that the high-frequency mode is 10 times weaker than the low-frequency mode (Fig. 4d), is provided in the first part (S.1) of the Supplementary information.

An ethanol and water droplet were simulated as a hemisphere with a 25 µm radius centered on the slot-dipole antenna’s gap between the transmission lines. The gap is 4 µm wide and is the place of the strongest electric field. During our investigations we have established that the detector’s signal value depends linearly on the volume of the applied material. The shape of the droplet, as well as the temperature variation in the microenvironment of the active area of the structure due to surface waves’ heat dissipation (plasmothermal effect^33^), are other very important parameters when designing a real-world dielectric sensor. Details on the model for ethanol and water dispersion of relative permittivity ($\epsilon = \epsilon ' - j\epsilon ''$) at THz frequencies can be found in the third part (S.3) of the Supplementary information. Both water and ethanol cause a red-shift in the resonance peak of the detector’s response, indicating a shift towards lower frequencies. Additionally, they attenuate the detected signal, leading to a decrease in signal strength. This effect is more pronounced with water compared to ethanol due to the larger values of both the real and imaginary parts of water’s permittivity within the frequency range of 100-500 GHz. At frequencies above the resonance, the simulations predict that the water-loaded sensor’s signal will get higher than the ethanol-loaded sensor’s signal, and even the same as the reference signal (see black, blue, green curves in the frequency range 400–500 GHz in Fig. 4a and black, blue, green curves around 500 GHz in Fig. 4b). The experiment findings somewhat deviate from this prediction (see Fig. 4c). In the same frequency range of 400–500 GHz, in which the 235 GHz resonance antenna displays a frequency flat impedance characteristic, there is an observable attenuation for both ethanol and water. One possible explanation for the disparity could be the difference in the amount of test liquids used in simulations and practical measurements.

For the simulations, we modeled a droplet shape of 33$\times 10^{-12}$ L volume (a hemisphere with a 25 µm radius) and reduced it to 2$\times 10^{-15}$ L volume (a hemisphere with a 1 µm radius). This minute volume of the load still produced a differential signal (see the third part (S.3) of the Supplementary information) approximately two orders of magnitude larger than the detector’s noise level. In contrast to the simulations, in the practical situation, we applied an amount of $\approx$ 4$\times 10^{-6}$ L. In the future, we intend to co-integrate a microfluidic channel and scale the volume of applied material down to reach the same order of the test liquids’ volume as in the simulations. Handling droplets of that size would enable access to single-cell manipulation^34^ as it is convenient using microfluidic platforms.

**Figure 5:**
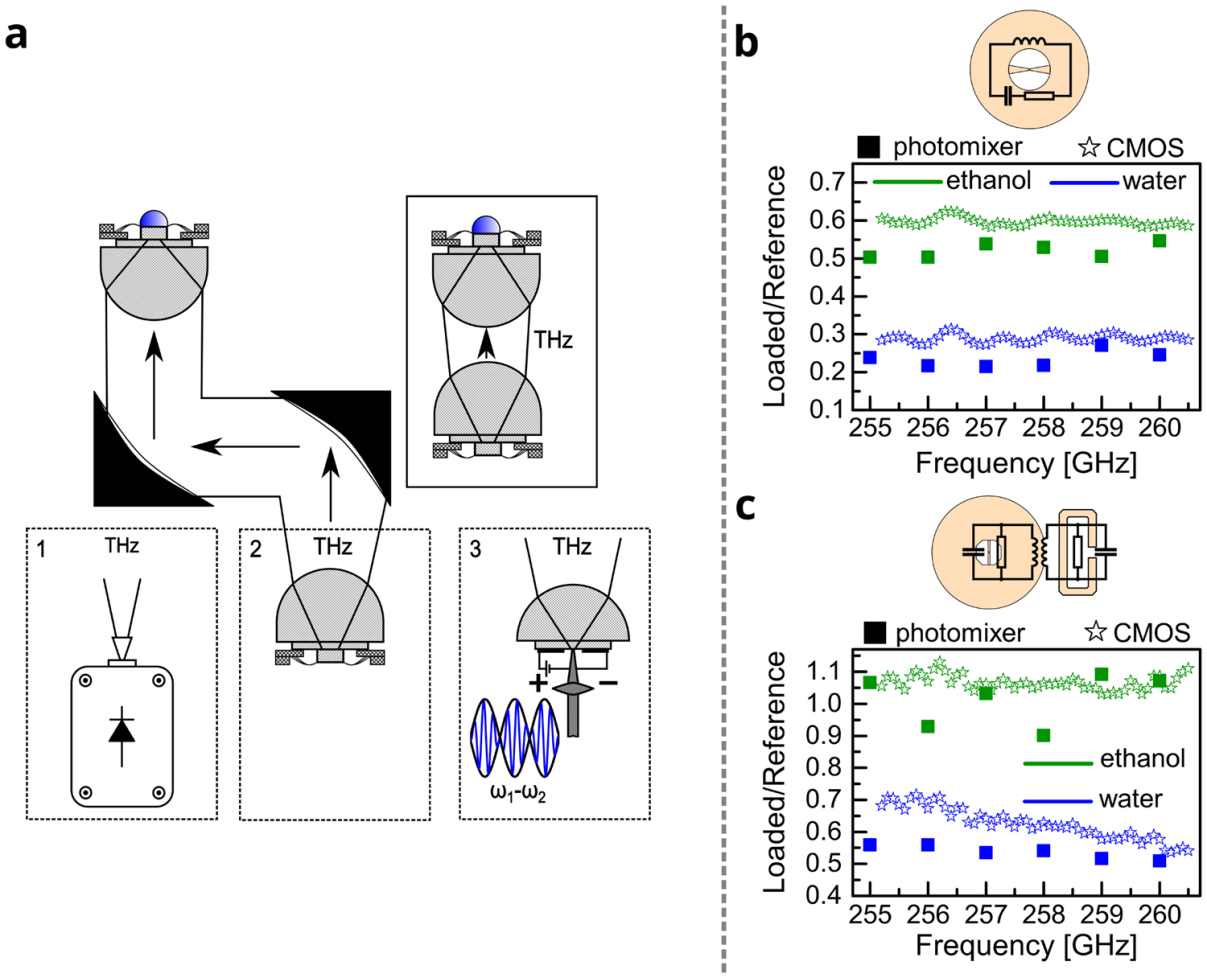
A setup schematic and results for the sensing experiment. (**a**) Sensing experiment with three possible sources of THz radiation and an illustration of a more compact experimental configuration. (**b**,**c**) Ethanol and water spectra probed with a widely tunable source based on photomixers and with a CMOS-based electronic source and recorded with a (**b**) 235 GHz resonance detector, (**c**) coupled resonators-based detector.

The THz source part required for the sensor can be further optimized. We present the results of the spectroscopy experiments conducted with an in-house designed CMOS-based THz emitter tunable in a frequency range of 248–261 GHz (Fig. 5). The emitter utilizes the concept of a Colpitts oscillator optimized for third harmonic emission. It was fabricated in a 65 nm CMOS technology provided by the TSMC foundry. More details on the emitter can be found in the following article^35^ (voltage-controlled oscillator number three, VCO3). In Fig. 5a an illustration of an experimental setup with an all-electronic CMOS-fabricated emitter-sensor pair (central configuration with a THz source number two) is shown.

In the same free-space quasi-optical setup, a continuous-wave photomixer source was used (THz source number three) to probe near-field dielectric properties. All experimental results prior to this discussion were obtained with the latter THz source (Fig. 3b–d). The last THz source (source number one) in the picture is a Schottky diode-based frequency multiplier. Highlighted by a solid rectangular box is the next step toward integration with direct emitter-sensor radiation coupling without additional optical components. It is still realized in free space though. The final step of integration would be emitter-sensor power coupling on-chip. As both the emitter and the sensor are fabricated in silicon semiconductor technology, power could be guided by silicon-on-insulator waveguides. The sensor’s response to ethanol and water loading in the frequency range 255–260.5 GHz is shown in Fig. 5b,c. Data points with a frequency step of 1 GHz in a scatter plot are essentially the same data points as in Fig. 4c,d but zoomed in. They were obtained with the terahertz photomixer source. Alongside them, data points obtained with the CMOS-based THz emitter are presented with a frequency step of 0.1 GHz. The sensor’s response is normalized by the power of the corresponding THz source and by the reference measurement of the detector with no load on the antenna. The finely tunable CMOS source allows getting a zoomed-in picture of the broad spectra recorded with the photomixer source. The coupled resonators-based detector has the responsivity maximum at $\approx$ 283 GHz while the other detector’s responsivity maximum is at 235 GHz. Thus, with the CMOS source, we probed the sample’s permittivity either at the low-frequency slope of the resonance curve ($\omega < \omega _0$) or at the high-frequency slope of the resonance curve ($\omega > \omega _0$).

**Figure 6:**
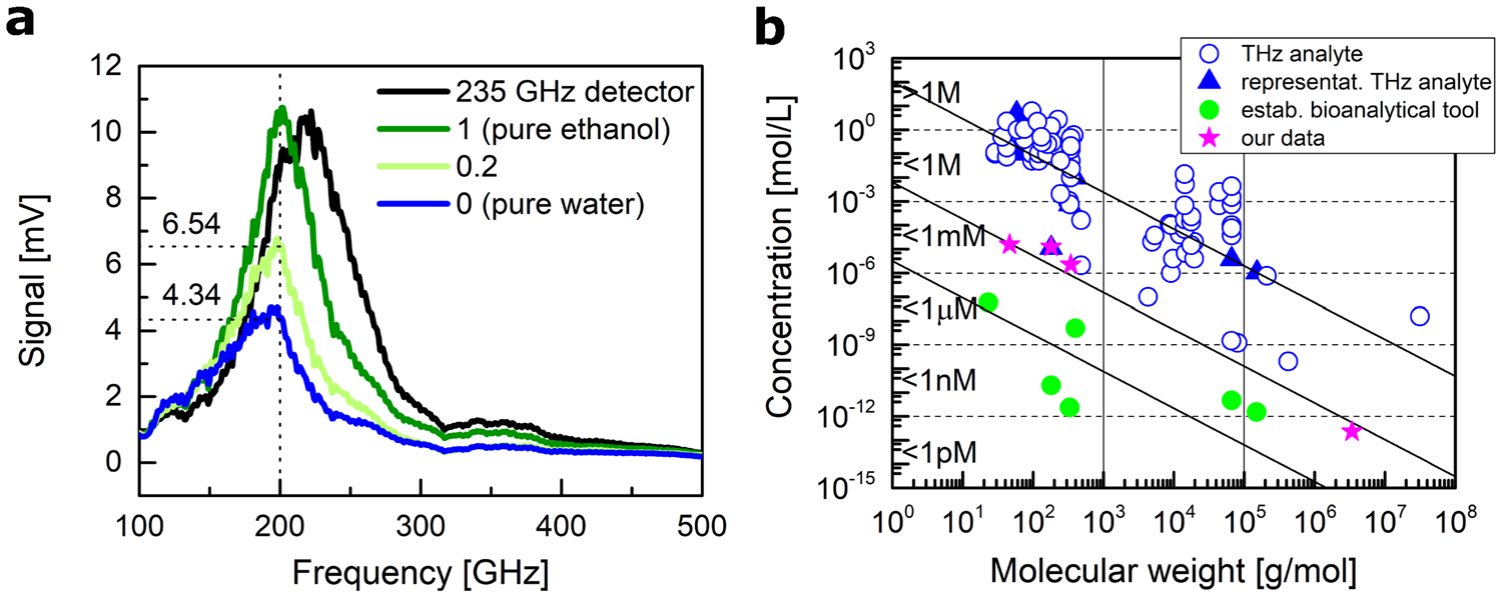
Results of the experimental measurement of an ethanol-in-water mixture and comparison to the state-of-the-art techniques. (**a**) Reference voltage response of the 235 GHz resonance detector and the same detector’s response loaded by pure ethanol, a 0.2 ethanol-in-water mixture, and pure water. (**b**) Our lower detection limit estimation alongside the reported LDL values for THz-based techniques and established bioanalytical tools^2^.

Finally, a set of experiments was performed with a 235 GHz resonance antenna-based detector to determine the lower detection limit (LDL) in terms of the lowest distinguishable molar concentration of material in an aqueous solution. Figure 6a presents spectra of the response in the 100-500 GHz frequency range loaded with pure water, pure ethanol, and an ethanol-in-water solution of 0.2 ethanol mass fraction. The limit for detection, therefore, is calculated from the experimental data. It is evaluated from the difference in signals (pure water and ethanol-in-water with 0.2 mass fraction) and related to the measured fluctuation spectral density of $10\times 10^{-9}~$V/$\sqrt{\textrm{Hz}}$. From this, we obtain the lower detection limit value of $1.56\times 10^{-5}~$[mol/L]. Similar sensitivity determination has been performed for solutions of glucose ($1.25\times 10^{-5}~$[mol/L]), sucrose ($2.21\times 10^{-6}~$[mol/L]), as well as for a plasmid with 5524 base pairs ($2.31\times 10^{-13}~$[mol/L]). Figure 6b demonstrates that values compare well to the extensive database of THz biomolecular sensing experiments performed in aqueous environments diligently gathered by the authors of the following review published in 2021^2^. According to this diagram, our sensor performs at the forefront of the developments in the field, contributing a relevant measurement result for lightweight molecules alongside the best results obtained for heavier molecules^36–40^.

## Conclusions

In this paper, we have reported the design and characterization results of the terahertz near-field resonator-based sensor, implemented using a 180 nm CMOS technology and featuring an antenna-integrated field-effect transistor. Operating on the principle of resonance curve shifts induced by changes in relative permittivity within the near-field of the resonator, our sensor offers a distinctive on-chip detection mechanism. Additionally, it showcases lower detection limit values at the forefront of current THz sensing techniques, achieved without any amplifications in sensitivity or specificity, such as conjugation mechanisms and polymerase chain reactions, in a label-free manner for an organic analyte in an aqueous environment. The results we have presented lay a solid foundation for several highly promising developments.

Firstly, while we have demonstrated proof-of-concept with currently available analytes and existing technological capabilities, simulations indicate the potential to scale down analyte volumes by six orders of magnitude while still maintaining competititve sensitivity levels. Secondly, the presented sensor, fabricated in standard CMOS technology, yielded promising results in spectroscopy measurements using an in-house designed state-of-the-art THz electronic source. Lastly, the concept of a coupled resonators-based near-field sensor, as presented in this work, introduces the potential for strong coupling of vertically arranged resonators. This innovation holds promise for sensing minute analyte concentrations and opens avenues for further exploration in sensor design.

In conclusion, our work not only contributes to the current understanding of terahertz near-field sensing but also sets the stage for the development of a high-frequency microfluidic lab-on-chip dielectric spectroscopy sensor.

## Methods

### CMOS chip design

A 180 nm CMOS technology was used to fabricate chips for the near-field sensor, and 65 nm CMOS technology was utilized for the voltage-controlled oscillator (VCO)-based THz radiation source. Both technologies belong to the Taiwan Semiconductor Manufacturing Company (TSMC) foundry. The VCO-based THz radiation source characterization can be found in^35,41,42^. The terahertz CMOS detector characterization is described in Refs.^43,44^. An additional $500~\upmu$m-thick silicon slab was attached under the chip for the silicon hyper-hemispherical lens ($\varnothing 12$ mm) positioning to improve free space radiation coupling in both the sensor and the radiation source. Each of the CMOS chips was wire-bonded on an individual printed circuit board (PCB). The chip for the near-field sensor, placed lower than the PCB, allowed us to create a well from glue to protect the bonds and leave open only the antenna area of $\approx$ $500\times 500~\upmu$m$^2$ large. This facilitated the accurate placement of droplets onto the active area of the sensor during the near-field sensing experiments. The PCB with the chip is mounted on an XY translation stage that provides precise alignment of the chip in relation to the fixed substrate lens (Fig. S5 in the fourth part (S.4) of the Supplementary information with more details in Ref.^35^). The XY mount is placed on a manual XYZ stage for the course alignment with the quasi-optical system. The near-field sensor is positioned in the plane parallel to the optical table, providing access to the chip’s sensitive area and the possibility of putting liquids on it.

### Measurement set-up

The measurement set-up is arranged as follows: A free space propagating Gaussian beam is collimated and focused by two 2$\;''$-f off-axis parabolic mirrors (OAP) with a focal distance of 3$\;''$. Both DC and RF measurements were prepared in the same quasi-optical setup. Additionally, we used Keithey 2400 source measure units as a voltage supply and for the current-voltage characterization. For low-noise signal measurements, TeraFETs were connected to a lock-in amplifier Ametek 7270. The modulation frequency for the carrier signals was $\approx$ 1 kHz. A commercial THz frequency multiplier radiation source based on Schottky diode with WR3.4 waveguide output from Virginia Diodes, Inc. and Frequency-Domain Terahertz Spectroscopy System, TeraScan 1550, TOPTICA Photonics AG were used in the experimental setup after the near-field sensing experiments with the VCO-based THz source as reference THz emitters covering a larger frequency range.

### Solution preparation and near-field sensing experiments methodology

Deionized water was used for all the near-field sensing experiments. The desired mass ratio for the mixtures was weighted. Proper mixing was ensured by utilizing a magnetic stirrer for 1 minute at 200 revolutions/minute at room temperature. Droplets of analytes with a volume of $4\times 10^{-6}$ L were delivered to the sensor with a micropipette. The curves in Figs. 4c,d, 5b,c and 6a, as well as experimental data for the lower detection limit estimation in 6b were averaged over 5 measurements. The standard near-field sensing experiment procedure included a series of reference measurements with no load on the antenna to establish the expected TeraFET’s operation. These were followed by a series of alternating measurements between an experiment with a load on the antenna (water or ethanol) and a reference experiment. When working with solutions, after ensuring the proper TeraFET’s behavior, the interchange between measurements with pure water and a particular solution (e.g. ethanol-in-water with 0.2 mass ratio) was performed for 5 consecutive times. Water, ethanol, or solutions were removed with lint-free anti-static wipes and the help of capillary action, as well as with a flow of compressed dry air. In the case of solutions, an additional step with pure water was added after the solution removal, not for the purpose of measurements but to ensure that no residual solution was left on the sensor. The signal value read from a lock-in amplifier served as a gauge of the sensor’s surface being ready for the next measurement. The statistical analysis is conducted to estimate noise spectral density and to calculate the mean signal from independent measurements.

Deionized water is produced on-site. We used sucrose, which is store-bought table sugar, in our experiments. We purchased 99% pure glucose powder and a pure ethanol solution from LLC WARCHEM. The plasmid was subcloned at Oliver Griesbeck’s laboratory at the Max-Planck-Institute for Biological Intelligence. The DNA solution was prepared as follows: the DNA coding for the mCyRFP2 fluorescent protein was cloned into the pCAGIG mammalian expression vector, resulting in a complete plasmid size of 5,524 base pairs. Plasmid preparation was performed in XL1-Blue chemically competent cells. The DNA was purified using the MACHEREY-NAGEL NucleoBond Xtra Midi kit for transfection-grade plasmid DNA and eluted in deionized water.

## Supplementary Information

Supplementary Information.

## Data Availability

Correspondence and requests for materials should be addressed to corresponding authors: Alexander Chernyadiev and Alvydas Lisauskas.
