# Publisher Correction: A CMOS-integrated terahertz near-field sensor based on an ultra-strongly coupled meta-atom

**DOI:** 10.1038/s41598-024-70968-5

**Published:** 2024-08-29

**Authors:** Alexander V. Chernyadiev, Dmytro B. But, Yurii Ivonyak, Kęstutis Ikamas, Alvydas Lisauskas

**Affiliations:** 1 CENTERA Laboratories, Institute of High Pressure Physics PAS, Sokołowska St. 29/37, 01-142 Warsaw, Poland; 2 Institute of Applied Electrodynamics and Telecommunications, Vilnius University, Saulėtekio Av. 9, 10222 Vilnius, Lithuania

Correction to: *Scientific Reports* 10.1038/s41598-024-61971-x, published online 20 May 2024

The Supplementary Information files published with this Article contained an error where the supplementary figures were published without their accompanying captions. The original Supplementary Information figures are provided below.

This error has now been corrected in the Supplementary Information file that accompanies the original Article.

### Supplementary Information

Supplementary Figure 1.

Supplementary Figure 2.

Supplementary Figure 3.

Supplementary Figure 4.

Supplementary Figure 5.

Supplementary Figure 6.

Supplementary Figure 7.

Supplementary Figure 8.

Supplementary Figure 9.

Supplementary Figure 10.

Supplementary Information.

Supplementary Figure 11.

