# Supplementary material for: A CMOS-integrated terahertz near-field sensor based on an ultra-strongly coupled meta-atom
Source: Sci Rep. 2024 May 20;14:11483. doi: 10.1038/s41598-024-61971-x (PMC11106299; doi:10.1038/s41598-024-61971-x)
Supplement: Supplementary file 1 — Supplementary Information. [file 41598_2024_61971_MOESM1_ESM.pdf]

Supplementary information

A CMOS-integrated terahertz near-field sensor based on an ultra-strongly coupled meta-atom

Alexander V. Chernyadiev [1*], Dmytro B. But [1], Yurii Ivonyak [1], Kęstutis Ikamas [2] and Alvydas Lisauskas [2*]

[1]CENTERA Laboratories, Institute of High Pressure Physics PAS, Sokołowska st. 29/37, Warsaw, 01-142, Poland.
[2]Institute of Applied Electrodynamics and Telecommunications, Vilnius University, Saulėtekio av. 9, Vilnius, 10222, Lithuania.

*Corresponding author(s). E-mail(s): acherniadev@unipress.waw.pl; alvydas.lisauskas@ff.vu.lt;
Contributing authors: dbut@unipress.waw.pl; yi@unipress.waw.pl; kestutis.ikamas@ff.vu.lt;

S.1 Electromagnetic modes of the ultra-strongly coupled meta-atom cell

Fig. S1 shows the radiation directivity pattern of the antenna-SRR coupled resonators at the frequencies of the two resonance modes: 274 GHz and 425 GHz. The simulation results reveal that the directivity pattern of the low-frequency resonance mode has a single main lobe, while the high-frequency resonance mode exhibits two major lobes. Thus, at 274 GHz, the coupled meta-atom cell behaves as a directional antenna, whereas at 425 GHz, a radiation pattern similar to that of a dual-beam antenna is created. This result explains the disparity between the numerical simulations in Fig. 4b and the experiment in Fig. 4d in the main manuscript. When the free space radiation illuminates the structures from the substrate's side with normal incidence, the Gaussian beam couples to the structures' radiation pattern almost optimally at the low-frequency resonance mode and poorly at the high-frequency resonance mode.

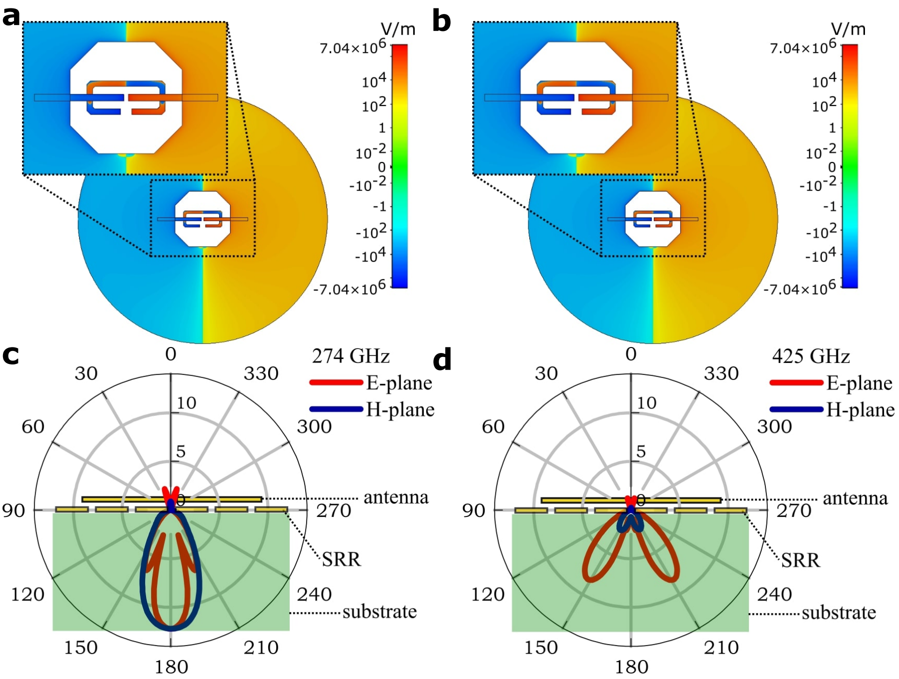

Fig. S1: Electromagnetic properties of the coupled resonators at two resonance modes. (a) Distribution of the normal component of the electric field $\vec{E}_n$ at 274 GHz. (b) Distribution of the normal component of the electric field $\vec{E}_n$ at 425 GHz. (c) Far-field radiation directivity pattern at 274 GHz. (d) Far-field radiation directivity pattern at 425 GHz.

S.2 Lumped-element model for the resonator's impedance

The impedance of an antenna can be calculated using the equivalent circuit model. The impedance characteristic of the slot-dipole antenna, with a resonance at 350 GHz, was fitted with a parallel resonance circuit (Fig. S2a), following Eq. 1.

$$Z = \frac{1}{\frac{1}{R} + \frac{1}{X_L} + \frac{1}{X_C}}, \tag{1}$$

where $X_L = j\omega L$ is the inductive reactance and $X_C = \frac{1}{j\omega C}$ is the capacitive reactance. R, L, C are the lumped-element parameters of the equivalent circuit for the antenna: resistance, inductance, capacitance. The angular frequency is denoted by ω. Fitting the antenna's impedance (the blue curve in Fig. 3a in the main manuscript) with Eq. 1 yields the following lumped-element parameters: $R = 1072.7\ \Omega, L = 1.04 \times 10^{-10}\ H, C = 1.99 \times 10^{-15}\ F$. The resonance frequency of the circuit can be found using Eq. 2.

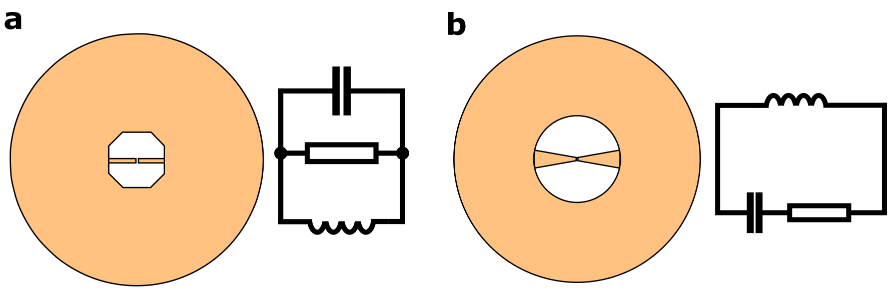

Fig. S2: Layouts and equivalent electronic circuits for (a) 350 GHz antenna and (b) 235 GHz antenna.

$$f_0 = \frac{1}{2\pi\sqrt{LC}} \tag{2}$$

The same model (Eq. 1) works very well for the split-ring resonator (the green curve in Fig. 3a in the main manuscript). The following lumped-element parameters were extracted after the fitting: $R = 1335.3\ \Omega, L = 6.66 \times 10^{-11}\ H, C = 3.1 \times 10^{-15}\ F$.

The antenna designed to resonate at 235 GHz (the dark yellow curve in Fig. 3b in the main manuscript) was described by a different equivalent schematic (Fig. S2b). A resistor was connected in series with a capacitor to simulate the antenna's flat response at higher frequencies, as expressed by Eq. 3.

$$Z = \frac{1}{\frac{1}{X_L} + \frac{1}{X_C + R}} \tag{3}$$

After fitting the impedance characteristic of the antenna resonant at 235 GHz, the following lumped-element parameters were obtained: $R = 46.05\ \Omega, L = 1.16 \times 10^{-10}\ H, C = 3.49 \times 10^{-15}\ F$.

One can use the equivalent schematic description for the system of coupled resonators too. We established that the mixed coupling model provides the most accurate fit to the mutual impedance of the slot-dipole antenna interacting with a split-ring resonator. In such a model there are two physical parameters linking the individual equivalent circuits: mutual inductance L_M and mutual capacitance C_M.

$$L_M = \kappa\sqrt{L_1 L_2}\frac{\sqrt{(1 + f_1^{-2} f^2)(1 + f_2^{-2} f^2)}}{2}, \tag{4}$$

$$C_M = \frac{C_1\kappa^2 + C_2\kappa^2 + \sqrt{(C_1\kappa^2 + C_2\kappa^2)^2 + 4\kappa^2 C_1 C_2(1 - \kappa^2)}}{2(1 - \kappa^2)} \times \\ \times \frac{\sqrt{(1 + f_1^{-2} f^2)(1 + f_2^{-2} f^2)}}{2}, \tag{5}$$

where $L_1, C_1, f_1, L_2, C_2, f_2$ are the inductance, the capacitance, and the resonance frequency of the first and the second equivalent RLC circuit, respectively. κ is the coupling coefficient representing the strength of the interaction. Frequency dependency of L_M and C_M [1] improves the description of the coupled resonators interaction over a large frequency range. The resonance frequencies f_1, f_2 are calculated from the well-known expression $f_{1,2} = \frac{1}{2\pi\sqrt{L_{1,2}C_{1,2}}}$ (as in Eq. 2). The overall impedance of the two coupled resonators can be calculated following the equivalent schematic in Fig. S3a step-by-step.

A good idea is to make a $\Delta-Y$ transformation of the three inductors connection. The expressions of the introduced inductors' parameters L_a, L_b, L_c are the following:

$$L_a = \frac{(L_1 - L_M)(L_2 - L_M) + L_M(L_1 - L_M) + L_M(L_2 - L_M)}{L_1 - L_M} \tag{6}$$

$$L_b = \frac{(L_1 - L_M)(L_2 - L_M) + L_M(L_1 - L_M) + L_M(L_2 - L_M)}{L_2 - L_M} \tag{7}$$

$$L_c = \frac{(L_1 - L_M)(L_2 - L_M) + L_M(L_1 - L_M) + L_M(L_2 - L_M)}{L_M} \tag{8}$$

Then, the mutual impedance calculation becomes straightforward. Equations 9 - 15 guide us toward the result.

$$Z_{R_2\|(C_2+C_M)} = \frac{R_2 \times \dfrac{1}{j\omega(C_2 + C_M)}}{R_2 + \dfrac{1}{j\omega(C_2 + C_M)}} \tag{9}$$

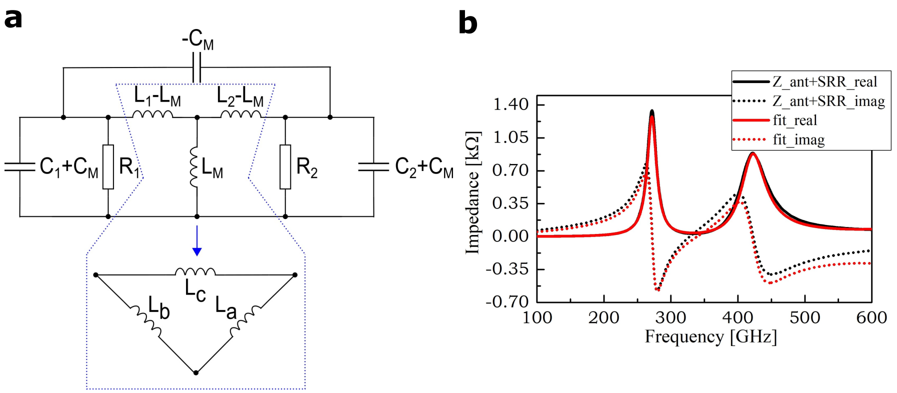

Fig. S3: Mixed coupling model for two resonance circuits. (a) The equivalent schematic. (b) The impedance characteristic of the slot-dipole antenna coupled to an SRR fitted by the mixed coupling model.

$$Z_{R_1\|(C_1+C_M)} = \frac{R_1 \times \dfrac{1}{j\omega(C_1+C_M)}}{R_1 + \dfrac{1}{j\omega(C_1+C_M)}} \tag{10}$$

$$Z_{L_c\|-C_M} = \frac{j\omega L_c \times \dfrac{1}{j\omega(-C_M)}}{j\omega L_c + \dfrac{1}{j\omega(-C_M)}} \tag{11}$$

$$Z_{Eq.\ 9\|L_a} = \frac{Z_{R_2\|(C_2+C_M)} \times j\omega L_a}{Z_{R_2\|(C_2+C_M)} + j\omega L_a} \tag{12}$$

$$Z_{Eq.\ 10\|L_b} = \frac{Z_{R_1\|(C_1+C_M)} \times j\omega L_b}{Z_{R_1\|(C_1+C_M)} + j\omega L_b} \tag{13}$$

$$Z_{Eq.\ 11+Eq.\ 12} = Z_{L_c\|-C_M} + Z_{R_2\|(C_2+C_M)\|L_a} \tag{14}$$

$$Z_{Eq.\ 14\|Eq.\ 13} = \frac{Z_{(L_c\|-C_M)+(R_2\|(C_2+C_M)\|L_a)} \times Z_{R_1\|(C_1+C_M)\|L_b}}{Z_{(L_c\|-C_M)+(R_2\|(C_2+C_M)\|L_a)} + Z_{R_1\|(C_1+C_M)\|L_b}} \tag{15}$$

The impedance of the slot-dipole antenna coupled to an SRR obtained from the numerical simulation was fitted with Eq. 15. The result is shown in Fig. S3b. The lumped-element parameters extracted from the fit are: $R_1 = 1642.59\ \Omega, L_1 = 7.08 \times 10^{-11}\ H, C = 3.97 \times 10^{-15}\ F, R_2 = 2481.75\ \Omega, L_2 = 5.55 \times 10^{-11}\ H, C_2 = 4.1 \times 10^{-15}\ F, \kappa = 0.16$.

S.3 Sensitivity limit

Sensitivity limit of the proposed near-field sensor can be estimated from the analytical model for a TeraFET detector [2, 3].

$$|\Delta V_{ref-loaded}| = -1/4 \frac{\partial \ln R_{ch}}{\partial V_g} f(\omega, \tau)[\Delta(H_V^2 8\Re(Z_{ant}))]\eta P_0, \tag{16}$$

where $|\Delta V_{ref-loaded}|$ is a differential, frequency- and gate-bias-dependent voltage signal induced in a TeraFET detector during the reference measurement, minus the voltage signal of the same detector but when loaded with a material. Placing a material on the antenna changes its impedance characteristic Z_{ant}; therefore, the only part that is different for the two measurements is highlighted by the square brackets in Eq. 16. $H_V 8\Re(Z_{ant})\eta P_0$ defines the voltage amplitude of the THz signal squared (V_{THz}^2) that is received by the antenna treated as an open-circuit voltage source V_a with maximum available optical power P_0. $H_V = |V_{THz}|/|V_a|$ is a voltage attenuation factor that determines the amplitude of the actual signal that reaches the transistor's channel. It is dependent on the antenna's impedance Z_{ant}. The rest of the expression does not change when the antenna is loaded. η is a parameter accounting for various sources of optical losses. $-\partial \ln R_{ch}/\partial V_g$ is the DC part of the analytical model for the transport

of charge carriers in a field-effect-transistor. $f(\omega, \tau)$ is a detection efficiency factor characterizing the propagation of charge density waves in the transistor's channel.

Looking deeper into the physics of the antenna's impedance change, we employed a straightforward approach using RLC equivalent circuits to model this change. In Appendix S.2, we introduced and described the RLC equivalents of two types of antennas, the SRR, and the mixed coupling model for the interaction between two resonators. At this point, it is important to recall that the material is placed in the proximity of the antenna's gap, the place with the strongest electric field. Merely from a very simple parallel plate capacitor model, one can see that inserting an ordinary dielectric material ($\epsilon > \epsilon_0$) with relative permittivity $\epsilon = \epsilon' - j\epsilon''$ into the capacitor's gap increases the capacitance ($C \sim \epsilon$) and introduces additional losses because of the imaginary part of the material's permittivity. Thus, an antenna loaded with a material can be viewed as the same equivalent circuit as for a plain antenna with no material but with a different resistance R and capacitance C.

In the realm of biosensing, the ability to accurately and sensitively detect and measure substances within water environments is of paramount importance. In this work, we tested our sensor's limitations with one of the fundamental organic compounds - ethanol. Thus, a set of numerical simulations was performed with a 33 pL droplet comprising varying ethanol-in-water ratios. Regarding the test liquids' dispersion at THz frequencies, the double Debye model was used for pure water, and the triple Debye model was used for pure ethanol [4]. The Bruggeman model [5] was applied to a mixture of two liquids. The extracted high-frequency impedance characteristics of both the stand-alone 235 GHz resonance antenna and the 350 GHz resonance antenna coupled to an SRR, loaded with such a droplet, were fitted with the corresponding equivalent schematic (the second part (S.2) of the Supplementary information). For this set of simulations, the antenna loaded with a droplet of pure water (ethanol fraction = 0) served as the reference measurement. The following lumped-element parameters were obtained after the fitting procedure of the reference measurement impedance curve: $R = 54.22\ \Omega, L = 1.16 \times 10^{-10}\ H, C = 4.26 \times 10^{-15}\ F$ according to Eq. 3 for the 235 GHz resonance antenna and $R_1 = 818.44\ \Omega, L_1 = 7.08 \times 10^{-11}\ H, C = 4.37 \times 10^{-15}\ F, R_2 = 2481.75\ \Omega, L_2 = 5.55 \times 10^{-11}\ H, C_2 = 4.1 \times 10^{-15}\ F, \kappa = 0.16$ according to Eq. 9 - 15 for the coupled resonators. Comparing these values to the ones presented in the second part (S.2) of the Supplementary information, one can notice that only R and C values of the antenna's equivalent schematic changed, whereas L value stayed fixed. The newly obtained resistance R and capacitance C for all chosen ethanol-in-water ratios are shown in Table St1 for the stand-alone antenna and in Table St2 for the antenna coupled to an SRR.

Resistance variation (col. 4 in Tables St1, St2) and capacitance variation (col. 5 in Tables St1, St2) as a function of ethanol mass fraction are shown in Figs. S4a-c. Resistance linearly changes for both the 235 GHz resonance antenna (Fig. S4a) and the 350 GHz antenna coupled to an SRR (Fig. S4b) when the mass fraction of ethanol in a droplet of water increases. The rate of capacitance change is different for the stand-alone resonator and the coupled resonators (Fig S4c). If the antenna is not coupled to another resonator, the capacitance increases linearly when more ethanol is present in a droplet of water (red square data points in Fig. S4c). However, in

Table St1: R and C parameters extracted from the equivalent schematic-based impedance fit of the 235 GHz resonance antenna loaded with ethanol-in-water mixtures

Ethanol mass fraction	R [Ω]	C [10^{-15} F]	| ΔR [Ω] |	| ΔC [10^{-15} F] |
0	54.22	4.26	0	0
0.05	53.86	4.22	0.36	0.04
0.1	53.46	4.19	0.76	0.07
0.2	52.58	4.12	1.64	0.14
0.25	52.09	4.08	2.13	0.18
0.33	51.24	4.03	2.98	0.23
0.4	50.52	3.99	3.7	0.27
0.5	49.43	3.93	4.79	0.33
0.6	48.36	3.88	5.86	0.38
0.7	47.36	3.83	6.86	0.43
0.8	46.45	3.79	7.77	0.47
0.9	45.65	3.75	8.57	0.51
1.0	45	3.71	9.22	0.55

Table St2: R_1 and C_1 parameters extracted from the equivalent schematic-based impedance fit of the coupled resonators loaded with ethanol-in-water mixtures

Ethanol mass fraction	R_1 [Ω]	C_1 [10^{-15} F]	| ΔR_1 [Ω] |	| ΔC_1 [10^{-15} F] |
0	818.44	4.37	0	0
0.05	844.79	4.3	26.35	0.07
0.1	866.76	4.28	48.32	0.09
0.2	915.13	4.25	96.69	0.12
0.25	941.63	4.235	123.19	0.135
0.33	989.12	4.21	170.68	0.16
0.4	1029.52	4.19	211.08	0.18
0.5	1095.08	4.16	276.64	0.21
0.6	1164.89	4.14	346.45	0.23
0.7	1236.24	4.11	417.8	0.26
0.8	1308.18	4.09	489.74	0.28
0.9	1376.89	4.07	558.45	0.3
1.0	1445.69	4.04	627.25	0.33

a coupled resonators system (green square data points in Fig. S4c) the capacitance increases at a quicker rate until the ethanol mass fraction reaches a certain point ($\approx$ 0.2), after which it grows slower than for a stand-alone antenna, following a square-root law. This brings us to a very important conclusion: for large variations in capacitance, a mere resonant antenna is more sensitive than an antenna coupled to another resonator. On the other hand, coupled resonators become more sensitive to smaller variations in capacitance. In other words, if we are probing a large change in dielectric properties, we would rather use a stand-alone resonant antenna, whereas, for fine changes in dielectric properties, coupled resonators are expected to show

better sensitivity. Finally, putting everything together, we can estimate the sensitivity

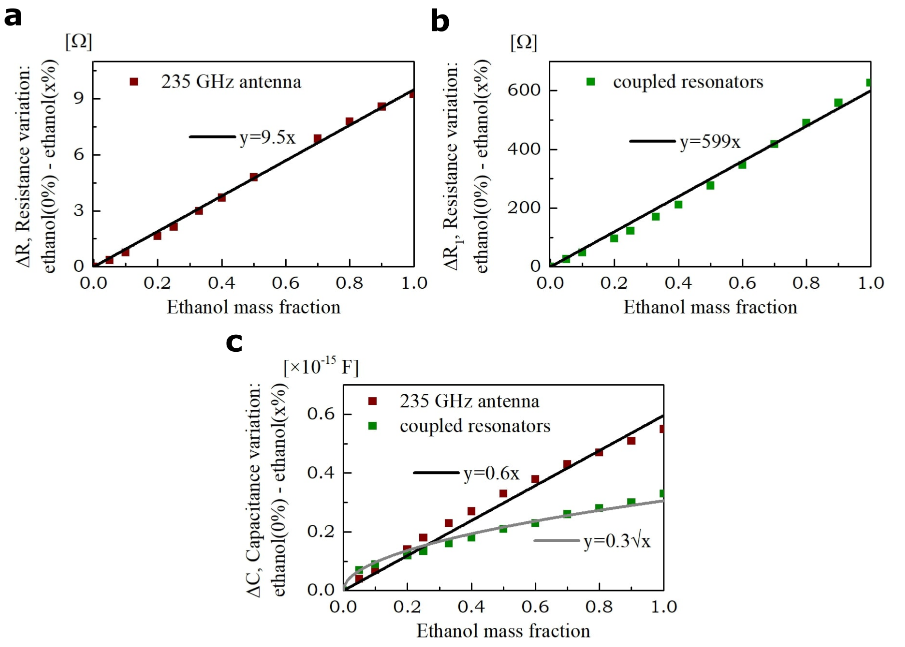

Fig. S4: Resonator's resistance and capacitance parameters change as a function of ethanol's mass fraction in ethanol-in-water mixture loading the sensor. (a) Resistance variation for 235 GHz antenna. (b) Resistance variation for coupled resonators. (c) Capacitance variation for both the 235 GHz antenna and the coupled resonators.

of our sensor using the analytical model for a TeraFET detector (Eq. 16), functions from Fig. S4c, and the noise level of the TeraFET detector at the operating point. At a 0.6 V gate bias, a field-effect transistor designed in the TSMC 180 nm process features a noise spectral density level of 10×10^{-9} V/$\sqrt{\text{Hz}}$. Next, by equating the left part of Eq. 16 to the transistor's noise level, we can find out what perturbation of the antenna's impedance Z_{ant} gives this level of the differential signal ΔV. We establish that an $10^{-5} - 10^{-6}$ order of perturbation in resistance and capacitance provides such a differential signal. Extrapolating the capacitance linear dependency from Fig. S4c in the 235 GHz resonance antenna to this level of perturbation brings us to 4.36×10^{-6} mass fraction of ethanol in water. Hence, the lower detection limit (LDL) in [mol/L]:

$$LDL = \frac{\rho}{M} W \frac{N}{\Delta V}, \tag{17}$$

where ρ is the component's density, M is the molar mass of the component, W is the component's mass fraction, N is the noise level of the detector. The LDL is expressed in terms of molar concentration or the smallest number of moles in a solution that

generates a measurable signal. Substituting the numbers for 4.36×10^{-6} ethanol-in-water solution leads to a lower detection limit of 6.26×10^{-5} [mol/L]. This is the estimation for a stand-alone resonance antenna-based detector. The same evaluation for the coupled resonators-based detector is more delicate and is the material for future work. If the same trend of a square-root law follows into the range of the tiniest mass fractions of ethanol (e.g. 6 orders of magnitude less than the values presented on a linear scale in Fig. S4c), then the LDL for such a detector will be exceptionally low. In order to verify such a claim, more data points have to be obtained in the range of $10^{-6} - 10^{-1}$ ethanol mass fraction.

S.4 Si CMOS chip packaging

Fig. S5 demonstrates the components comprising a CMOS-based terahertz detector module. The details are provided in [6].

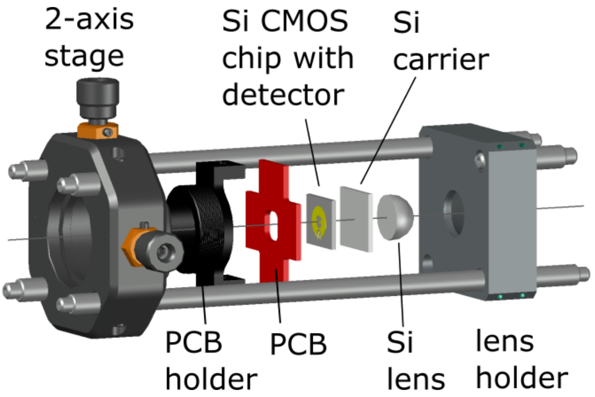

Fig. S5: An illustration of the CMOS-based terahertz detector module packaging.

Supplementary References

[SR1] Tyurnev V. The coupling coefficients of an asymmetric pair of microwave resonators. Journal of Communications Technology and Electronics, 47(1):1–8, 2002.

[SR2] Zdanevičius J., Čibiraitė D., Ikamas K., Bauer M., Matukas J., Lisauskas A., Richter H., Hagelschuer T., Krozer V., Hübers H.-W., and Roskos H. G. Field-effect transistor based detectors for power monitoring of THz quantum cascade lasers. IEEE Transactions on Terahertz Science and Technology, 8(6):613–621, 2018.

[SR3] Ikamas K., Čibiraitė D., Lisauskas A., Bauer M., Krozer V., and Roskos H. G. Broadband terahertz power detectors based on 90-nm silicon CMOS transistors

with flat responsivity up to 2.2 THz. IEEE Electron Device Letters, 39(9):1413–1416, 2018.

[SR4] Jepsen P. U., Møller U., and Merbold H. Investigation of aqueous alcohol and sugar solutions with reflection terahertz time-domain spectroscopy. Optics Express, 15(22):14717–14737, 2007.

[SR5] Bruggeman D. A. G. Berechnung verschiedener physikalischer Konstanten von heterogenen Substanzen. I. Dielektrizitätskonstanten und Leitfähigkeiten der Mischkörper aus isotropen Substanzen. Annalen der Physik, 416(7):636–664, 1935. [In German].

[SR6] Ikamas K., But D.B., Cesiul A., Kołaciński C., Lisauskas T., Knap W., and Lisauskas A. All-electronic emitter-detector pairs for 250 GHz in silicon. Sensors, 21(5795):1–19, 2021.
