# Supplementary figures and images for: Publisher Correction: A CMOS-integrated terahertz near-field sensor based on an ultra-strongly coupled meta-atom
Source: Sci Rep. 2024 Aug 29;14:20068. doi: 10.1038/s41598-024-70968-5 (PMC11362170; doi:10.1038/s41598-024-70968-5)

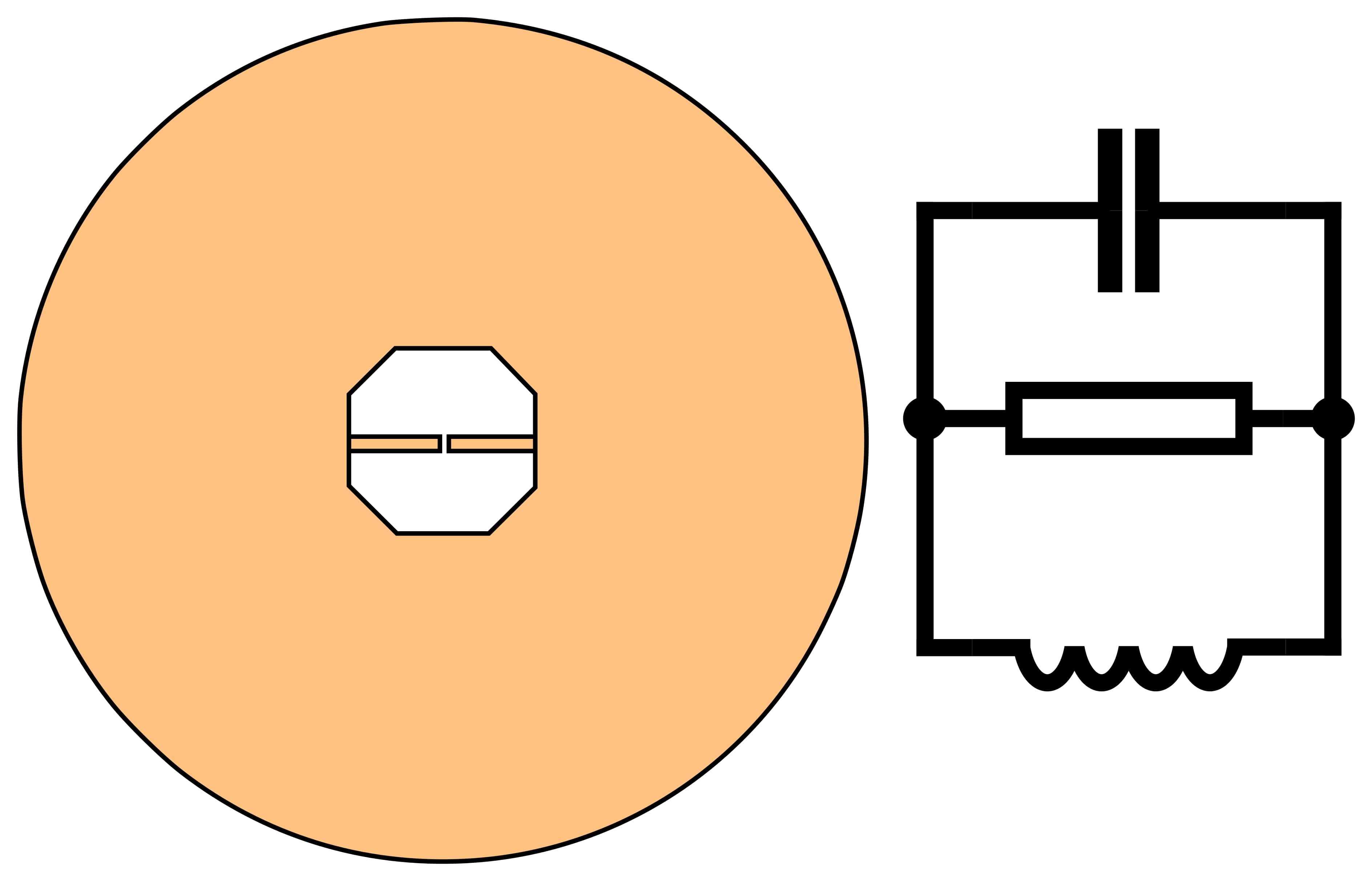

Supplement: Supplementary file 1 — Supplementary Figure 1. [file 41598_2024_70968_MOESM1_ESM.jpg]

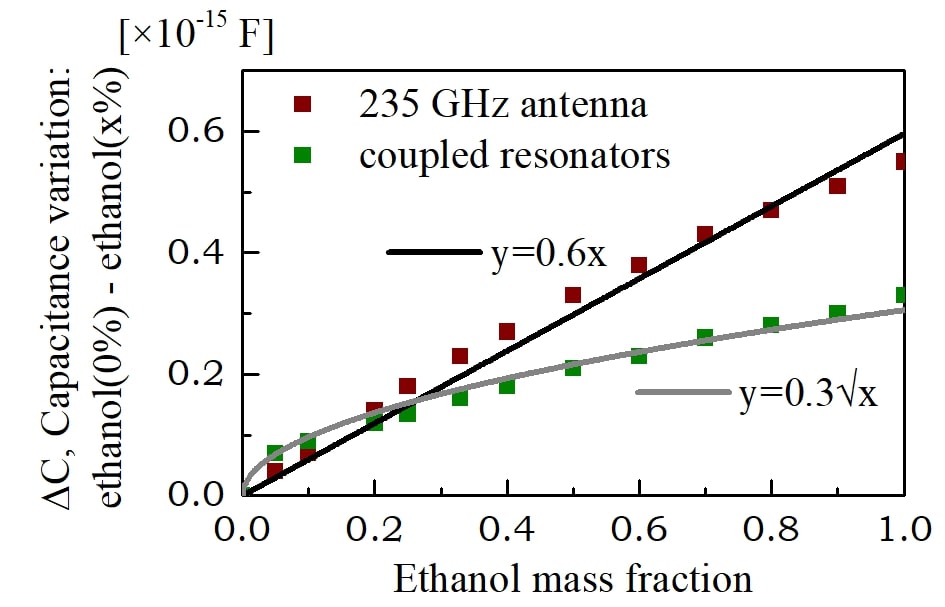

Supplement: Supplementary file 2 — Supplementary Figure 2. [file 41598_2024_70968_MOESM2_ESM.jpg]

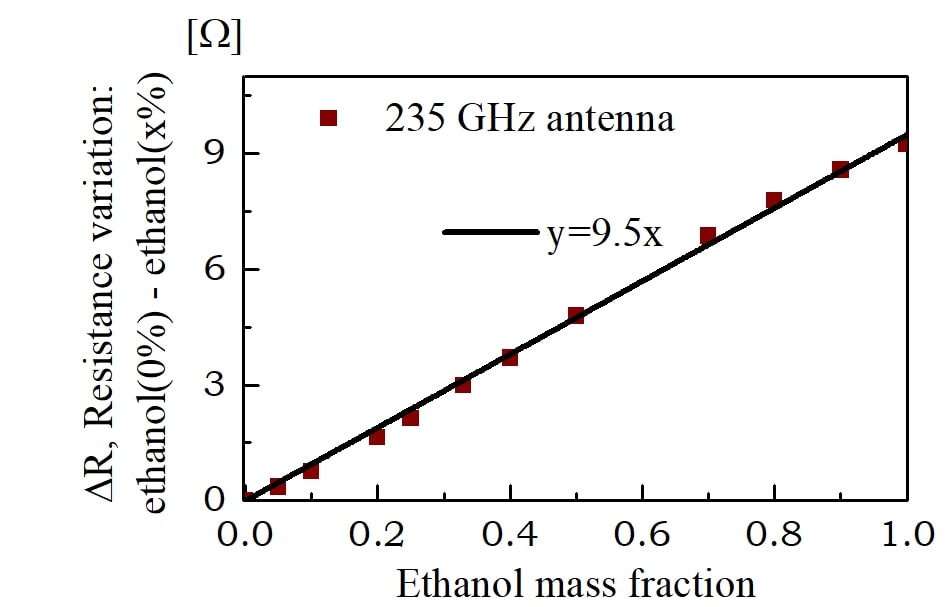

Supplement: Supplementary file 3 — Supplementary Figure 3. [file 41598_2024_70968_MOESM3_ESM.jpg]

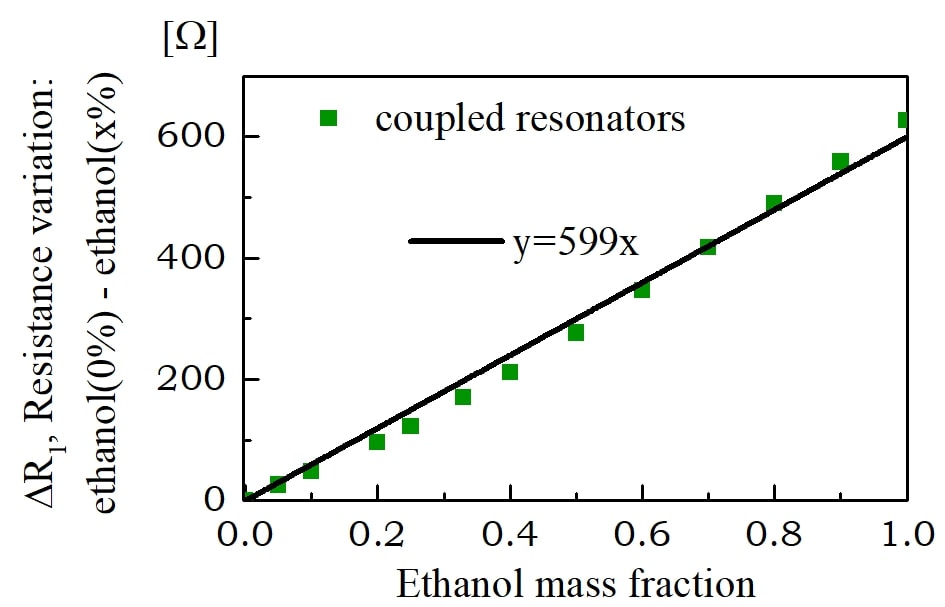

Supplement: Supplementary file 4 — Supplementary Figure 4. [file 41598_2024_70968_MOESM4_ESM.jpg]

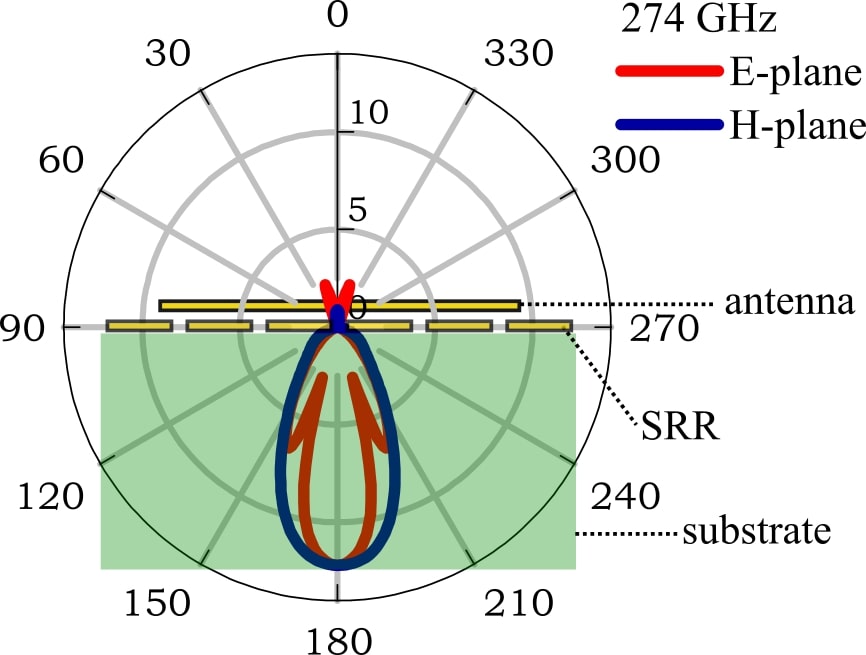

Supplement: Supplementary file 5 — Supplementary Figure 5. [file 41598_2024_70968_MOESM5_ESM.jpg]

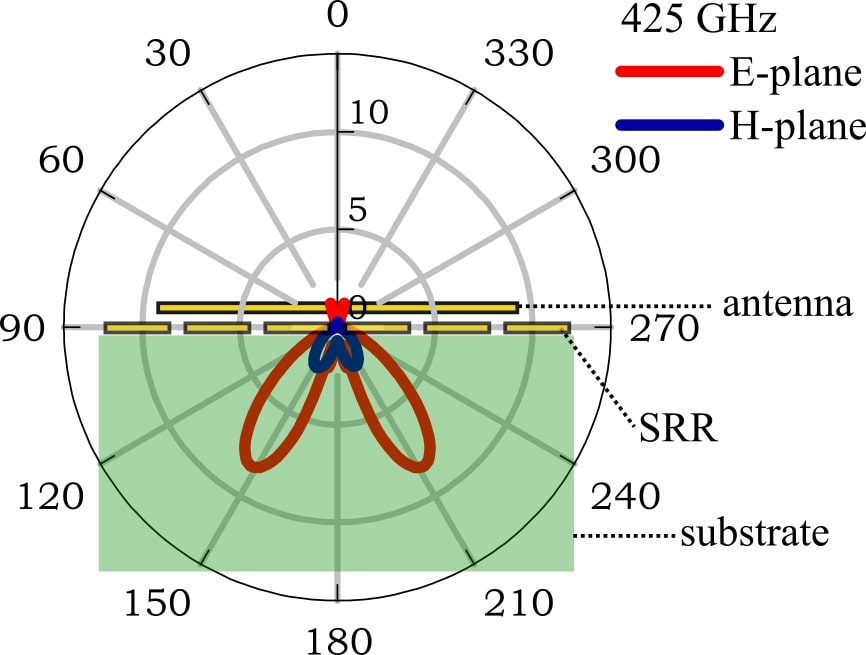

Supplement: Supplementary file 6 — Supplementary Figure 6. [file 41598_2024_70968_MOESM6_ESM.jpg]

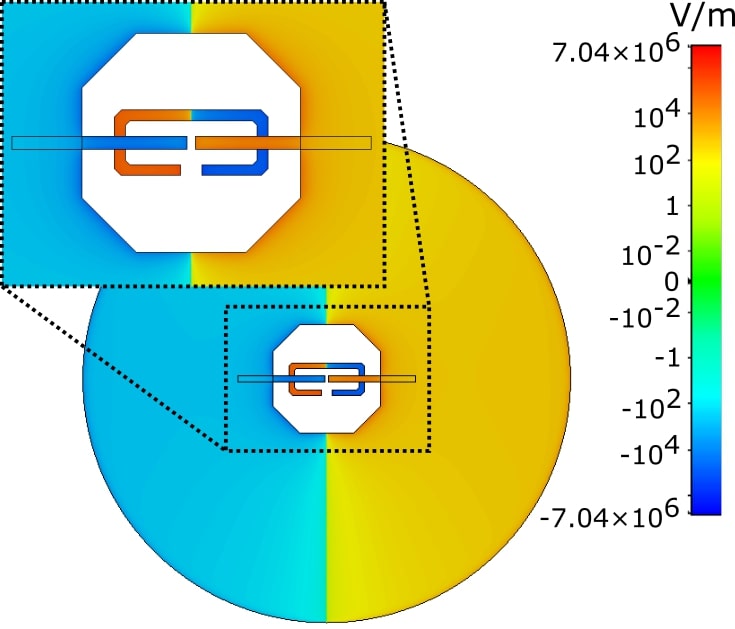

Supplement: Supplementary file 7 — Supplementary Figure 7. [file 41598_2024_70968_MOESM7_ESM.jpg]

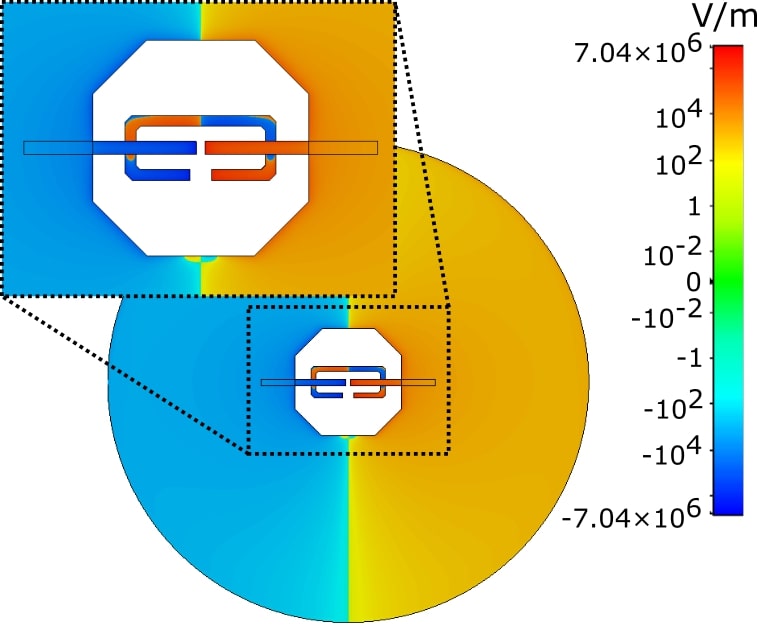

Supplement: Supplementary file 8 — Supplementary Figure 8. [file 41598_2024_70968_MOESM8_ESM.jpg]

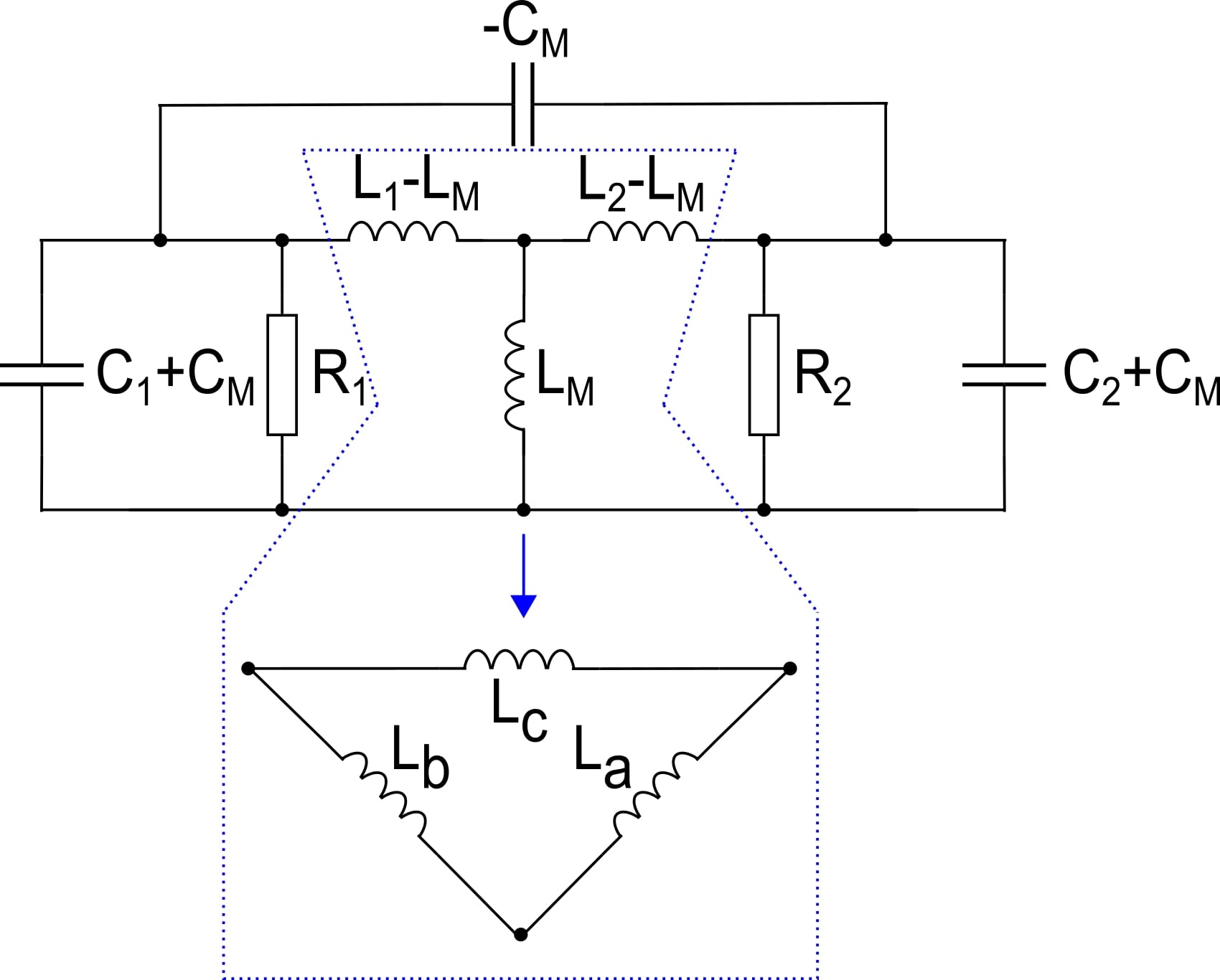

Supplement: Supplementary file 9 — Supplementary Figure 9. [file 41598_2024_70968_MOESM9_ESM.jpg]

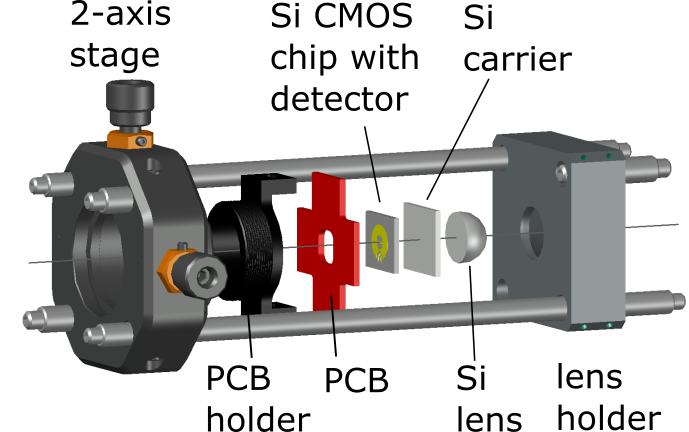

Supplement: Supplementary file 10 — Supplementary Figure 10. [file 41598_2024_70968_MOESM10_ESM.png]

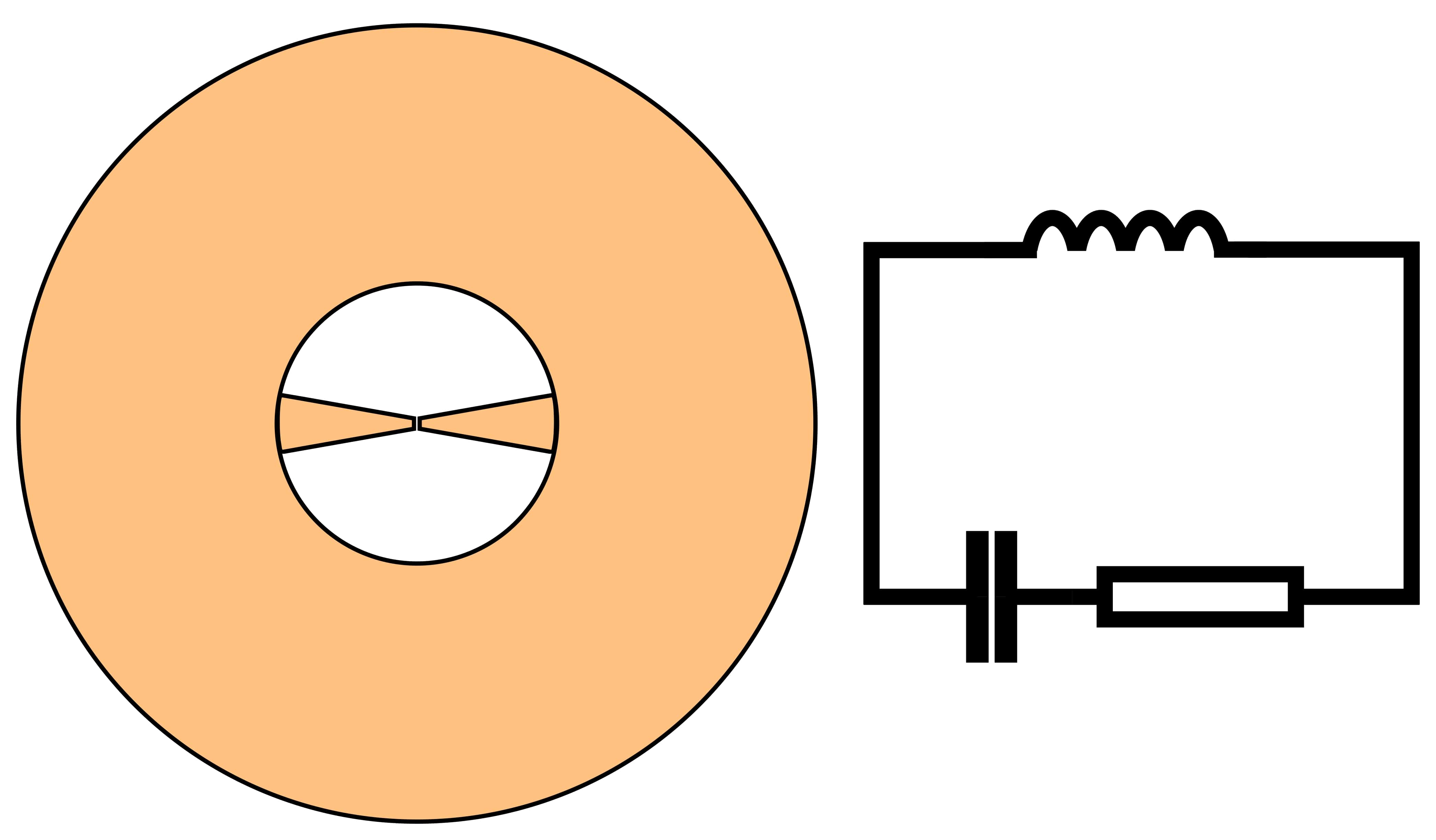

Supplement: Supplementary file 12 — Supplementary Figure 11. [file 41598_2024_70968_MOESM12_ESM.jpg]
